# Elevated Anti-Cytomegalovirus IgG Levels Are Associated with Higher Erythropoiesis-Stimulating Agent Dose in Transplant-Listed Patients with End-Stage Kidney Disease

**DOI:** 10.3390/jcm15218541

**Published:** 2026-11-02

**Authors:** Alexander Jerman, Miha Arnol, Vanja Peršič

**Affiliations:** 1 Department of Nephrology, University Medical Center Ljubljana, 1000 Ljubljana, Slovenia; miha.arnol@kclj.si (M.A.); vanja.persic@kclj.si (V.P.); 2 Faculty of Medicine, University of Ljubljana, 1000 Ljubljana, Slovenia; 3 Sferogen, 5270 Ajdovscina, Slovenia

**Keywords:** renal anemia, cytomegalovirus, erythropoietin, kidney disease

## Abstract

**Background**. CMV seropositivity has been associated with increased epoetin demand in patients with end-stage kidney disease (ESKD) undergoing kidney replacement therapy (KRT). However, the available data remain inconclusive. **Methods**. In this single-center retrospective study we screened adult kidney transplant candidates with dialysis-dependent ESKD and whose CMV serostatus was known. Baseline patient characteristics and erythropoiesis-stimulating agent (ESA) prescription data were evaluated. Multivariate analysis was performed to determine whether CMV seropositivity or anti-CMV immunoglobulin levels are associated with more frequent ESA prescription and higher standardized ESA weekly dose. **Results**. A total of 198 patients were included, with a median age of 51 years; 31% were female. The majority of patients (73%) were treated with hemodialysis, and the median dialysis vintage was 1 year. ESA therapy was prescribed to 77% of patients, with a standardized median weekly dose of 61 units per kilogram of body weight. CMV seropositivity was identified in 79% of patients and was associated with older age, longer dialysis vintage, higher serum CRP and ferritin levels, and lower serum total iron-binding capacity. In adjusted regression models, ESA prescription rate and ESA weekly dose were not associated with CMV seropositivity. Conversely, higher ESA weekly dose was associated with CMV IgG levels in the 5th quintile (both *p* < 0.001) after adjustment for hemoglobin levels and body iron stores. **Conclusion**. CMV seropositivity was not associated with an increased likelihood of ESA prescription among transplant-listed individuals with ESKD. In a fully adjusted regression model, however, standardized weekly ESA dose was significantly higher in individuals with anti-CMV IgG levels in the highest quintile. Further studies are warranted to determine the true nature of these observations. If confirmed, it will be important to clarify whether elevated anti-CMV IgG levels reflect underlying immune dysregulation, or conversely, whether CMV reactivations contribute both to increased IgG levels and to ESA hyporesponsiveness.

## 1. Introduction

Human cytomegalovirus (CMV) is highly prevalent in the general population. Although typically asymptomatic, latent CMV infection has been associated with increased morbidity (including immunosenescence [1,2,3,4], frailty [5], and atherosclerosis [6,7,8,9,10,11,12,13,14]) and mortality [15,16,17,18,19,20,21,22], but these observations remain inconclusive [23,24,25,26,27,28,29]. In patients with end-stage kidney disease (ESKD), CMV seropositivity has also been associated with more pronounced alterations in immune status [30] and other adverse changes [30,31,32,33,34,35,36,37,38,39]. It has been shown that CMV-specific immunoglobulin G (IgG) levels may serve as a marker of morbidity and immune dysregulation in the context of latent CMV infection [7,8,9,17,32,40,41]. Importantly, high anti-CMV IgG levels do not necessarily indicate an active CMV infection, but they might be associated with higher reactivation risk [42].

Patients with ESKD develop renal anemia as a consequence of impaired erythropoietin production. As an interesting exception, erythropoietin production is maintained in patients with polycystic kidney disease [43,44,45]. However, the ESA dose required to achieve a therapeutic effect is highly variable and is influenced by inflammatory status and other factors, including iron deficiency, chronic hemolysis, and concomitant medication [46]. Despite the numerous reported associations between proinflammatory milieu (e.g., C-reactive protein (CRP), interleukin-6 (IL-6), hepcidin, etc.), the treatment of renal anemia remains a challenging clinical issue.

Butler et al. provided evidence that CMV infection directly interferes with erythropoietin production and is associated with renal anemia [47]. Betjes et al. further demonstrated that CMV-seropositive patients with ESKD exhibited elevated epoetin demand, while pre-dialysis patients showed reduced hemoglobin levels [48]. In contrast, an observational cohort assessed by Baldeo et al. failed to replicate these observations in patients with chronic kidney disease (CKD) stage 5. Moreover, in a subgroup analysis of patients on hemodialysis, CMV seropositivity was associated with higher hemoglobin levels and reduced ESA dose [49].

The objective of the present analysis was to clarify the discordant findings regarding the association between CMV seropositivity and ESA use in ESKD patients. We conducted a retrospective cohort analysis to assess whether CMV seropositivity and anti-CMV specific immunoglobulin levels are associated with more frequent ESA prescription or higher ESA doses.

## 2. Materials and Methods

### 2.1. Aim

The aim of this study was to evaluate the association between CMV seropositivity and CMV-specific immunoglobulin G and the prescription and dose of ESA in a single-center cohort of patients with end-stage kidney disease (ESKD) listed for renal transplantation. This was a retrospective, single-center observational study.

### 2.2. Study Population

The study screened patients with ESKD on dialysis (peritoneal and hemodialysis) who were eligible for kidney transplantation and were referred to or treated at the Department of Nephrology at the University Medical Center Ljubljana, Slovenia, between 2000 and 2024. The center serves as the national referral center for kidney transplantation, covering approximately two million inhabitants. Patients with available CMV serostatus, ESA weekly dose, and key laboratory parameters (hemoglobin, ferritin) were included. Those under the age of 18 years and patients with a history of prior solid organ transplantation were excluded.

### 2.3. Data Retrieval

Basic patient characteristics were retrieved from electronic health records. Data were accessed between 08.01.2024 and 01.06.2024. Positive CMV serostatus was determined using a peripheral blood CMV-specific immunoglobulin G (IgG; DiaSorin Liaison^®^ CMV IgG (Diasorin, Saluggia, Italy)) test. Two distinct assays were used historically at this institution to measure anti-CMV IgG levels, precluding continuous modeling. Values at the limits of detection were not transformed and were coded as numerical values of the respective limit. Absolute CMV IgG levels were then categorized into quintiles according to the underlying distribution for each assay. The calibration curves for the two assays were not available for comparison. The boundaries for the quintiles were delineated as follows: [21.3, 85.5], (85.5, 100], (100, 124], (124, 151], and (151, 180] for the first assay, and [0.68, 4.78], (4.78, 5.92], (5.92, 10.8], (10.8, 19.2], and (19.2, 22] for the second assay. A comprehensive set of patient-related parameters was recorded at the time of CMV serostatus testing, including body weight, height, BMI, smoking status (based on self-reported smoking history), atherosclerosis (defined as a documented history of atherosclerotic disease prior to CMV testing), diabetes, primary kidney disease (as coded in the electronic health record), and autosomal dominant polycystic disease (ADPKD, coded when the primary kidney disease was recorded as “polycystic” or “ADPKD”). The weekly ESA dose, recorded within close proximity (0 to 3 months) to CMV IgG testing, was standardized across agent types. Darbepoetin was converted using the formula: units = micrograms × 200. Continuous Epoetin Receptor Activator (CERA) is not directly convertible; standardization was therefore based on average starting conversion using the formula: units = CERA (monthly units)/200 × 12,000. This approach yielded estimates comparable to those previously published [49], with slight underestimation at the lower end and slight overestimation at the upper end of CERA dose. The standardized weekly ESA dose was obtained by dividing the weekly ESA dose by the pre-dialysis body weight in kilograms.

### 2.4. Data Analysis

Data retrieved from electronic health records were organized using Microsoft Excel. After integration of the disparate datasets, all data were anonymized. The data manipulation and statistical analyses were performed using R (version 4.5.2, 2025) [50], with the following packages: readxl, tidyverse, gtsummary, Cairo, mice, and patchwork. Binary parameters were compared using the chi-square test or Fischer’s exact test; continuous parameters with normal and non-normal distributions were compared using the t-test and Mann–Whitney test, respectively. Continuous parameters were assessed for normality with the Kolmogorov–Smirnov test. Missing data for smoking history and diabetes status were imputed with predictive mean matching prior to inclusion in the regression models (m = 20 imputed datasets, 10 iterations). Estimates were pooled with Rubin’s rules. ESA prescription was modeled using logistic regression, and ESA dose was modeled using linear regression model. All available confounders were included in the adjusted models; interactions between covariates were not assessed. A two-sided *p*-value of less than 0.05 was considered statistically significant. Due to exploratory nature of the study, the multiple-testing corrections were not implemented. Power analysis was not conducted, as all patients at our institution who fulfilled the inclusion criteria were considered for inclusion. The robustness was assessed with the following: Cook’s distance was calculated for every participant in each imputed dataset; this was classified as influential if Cook’s distance was greater than 4/n (*n* = size of dataset) in more than half of the imputations. Regression models were repeated with the same covariates and compared to the initial dataset.

## 3. Results

### 3.1. Patient Characteristics

Baseline patient characteristics are presented in Table 1. The study comprised 198 patients with a median age of 51 years, of whom 31% were female. IgA nephropathy was the most common underlying cause of kidney failure, accounting for 23% of cases. The prevalence of smoking was 45%, while diabetes and atherosclerosis were both present in 18% of patients, respectively. The majority (73%) were treated with hemodialysis, with median dialysis vintage of 1 year. ESA therapy was prescribed in 79% of patients, with a median standardized weekly ESA dose of 61 units/week/kg.

**Figure A1:**
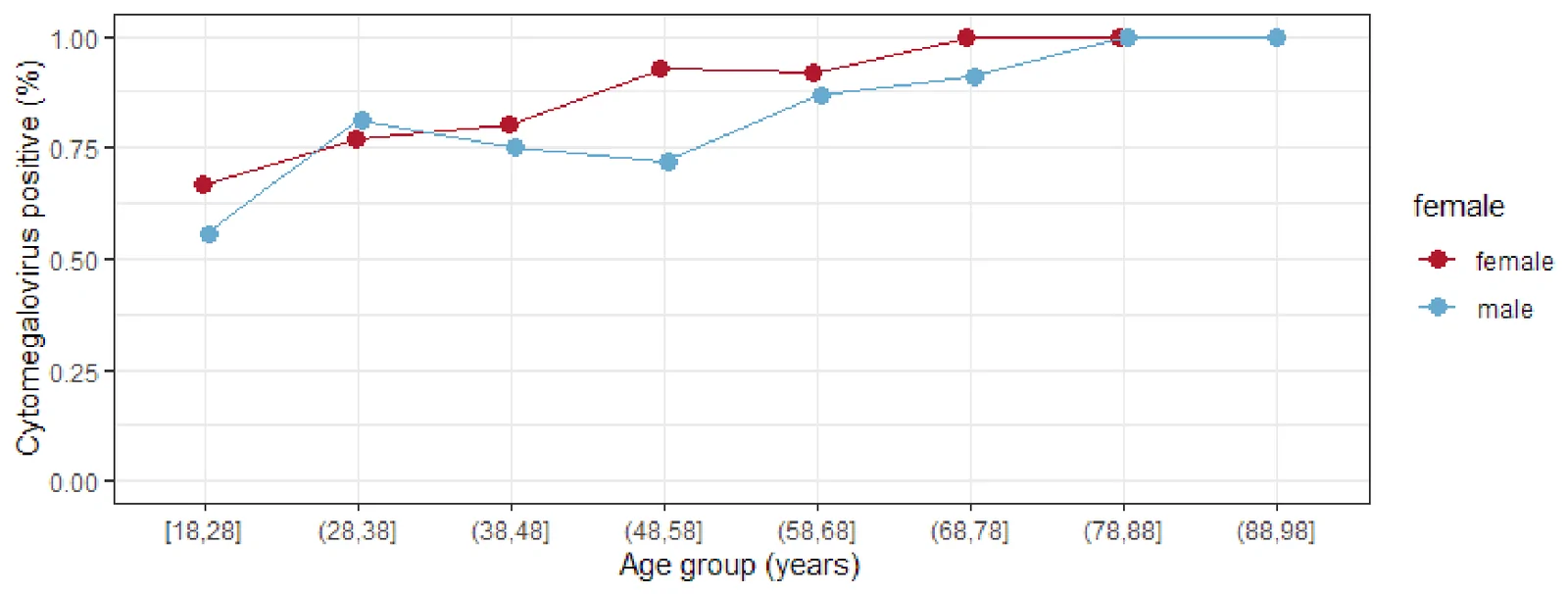
Cytomegalovirus prevalence according to the age group.

The overall CMV seroprevalence was 79%, with an age-dependent trend observed (see Appendix A, Figure A1). CMV-seropositive patients had longer dialysis vintage (1.20 vs. 0.55 years, *p* = 0.011), a tendency for higher serum CRP and ferritin levels, and lower serum total iron-binding capacity (TIBC).

### 3.2. CMV Seropositivity Is Not Associated with ESA Prescription

In exploratory analysis, no association was observed between ESA prescription rate and CMV seropositivity. Also, ESA weekly dose did not differ between groups. In order to improve our understanding of possible confounding, we modeled the ESA prescription in a generalized regression model. In the univariate model, the presence of ADPKD and higher hemoglobin was associated with lowered odds ratio (OR) for ESA prescription. The two multivariate models were adjusted for sex, age, atherosclerosis, diabetes, smoking, RRT vintage, RRT type, ADPKD, hemoglobin, CRP, ferritin, TIBC, and iron levels. The first multivariate model showed no association between CMV seropositivity and ESA prescription. The second model indicated a significantly elevated risk for ESA prescription in the group with anti-CMV IgG in the lowest quintile. In both adjusted models, higher hemoglobin levels were associated with lower ESA prescription rate, whereas atherosclerosis was associated with lower and diabetes with higher odds ratio for ESA prescription (see Appendix A, Table A1).

### 3.3. Higher Anti-CMV IgG Are Associated with Higher ESA Weekly Dose

**Figure 1:**
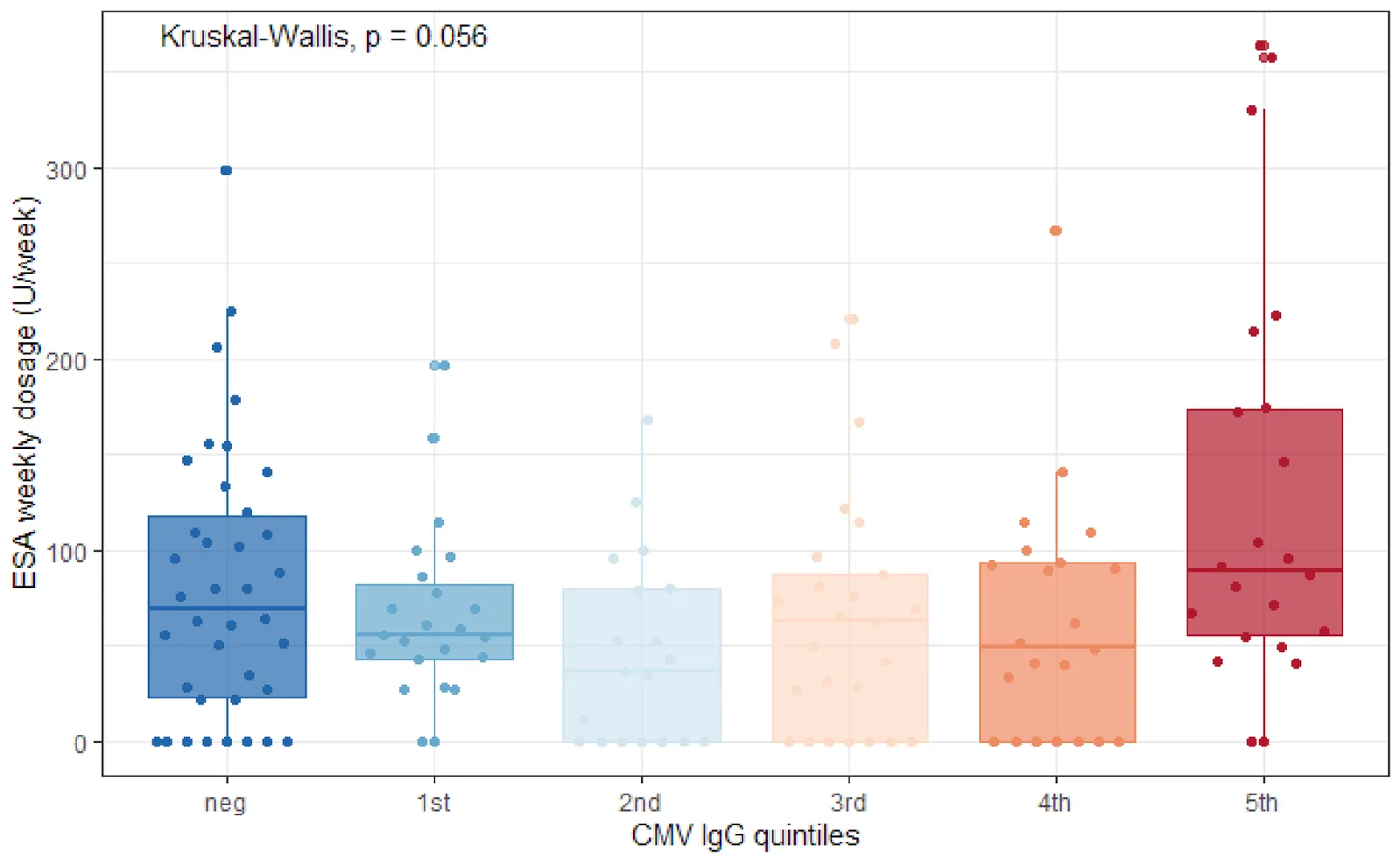
Erythropoiesis-stimulating agent (ESA) standardized weekly dose among patients with ESKD, stratified by CMV IgG quintiles. Rates across multiple groups were compared using the Kruskal–Wallis test. ESKD = end-stage kidney disease; CMV = cytomegalovirus; IgG = immunoglobulin G.

Two distinct anti-CMV IgG assays were used. The first was used on 29 and the second on 162 individuals. Eight individuals were found to have missing data concerning their anti-CMV IgG levels. Stratification by anti-CMV IgG quintiles revealed significant differences in age, prevalence of diabetes, dialysis vintage, and serum TIBC levels. Also, a borderline-significant trend towards higher ESA weekly dose, associated with higher serum anti-CMV antibody quintiles, was noted (see Table 2 and Figure 1).

We modeled the standardized ESA weekly dose initially using a univariate model, and subsequently with multivariate models adjusted for sex, age, smoking history, diabetes, atherosclerosis, RRT vintage, RRT type, ADPKD, hemoglobin, CRP, and ferritin levels. The covariates were selected based on a previous model by Betjes et al. (missing covariates: parathyroid hormone, ethnicity, serum albumin, and transferrin saturation). Additionally, we included atherosclerosis and ADPKD status, which are known to be associated with epoetin requirements (see Introduction). Since all subjects with missing anti-CMV IgG quintile (n = 8) were also missing body weight data (and thus standardized ESA weekly dosage), we omitted those subjects from further analysis.

**Figure 2:**
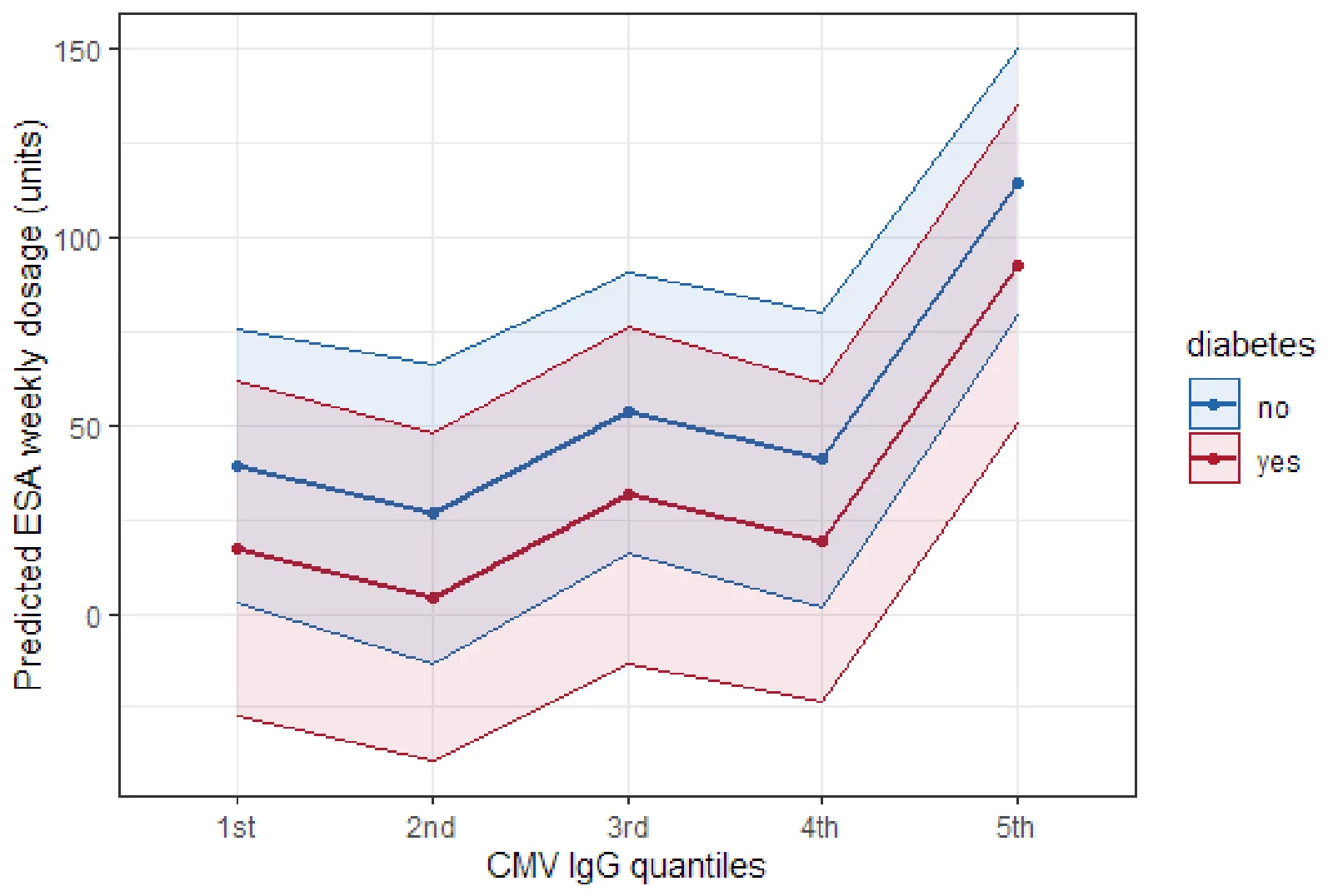
Predicted erythropoiesis-stimulating agent (ESA) standardized weekly dose from the multivariate adjusted model, stratified by the presence of diabetes. The prediction model was based on the fixed parameters, including female sex, age of 60 years, absence of atherosclerosis, smoking, and ADPKD, one-year RRT vintage, hemodialysis as RRT modality, hemoglobin 120 g/L, CRP 5 mg/L, and ferritin 200 μg/L. ESA = erythropoiesis-stimulating agent; CMV = cytomegalovirus; IgG = immunoglobulin G.

**Figure A2:**
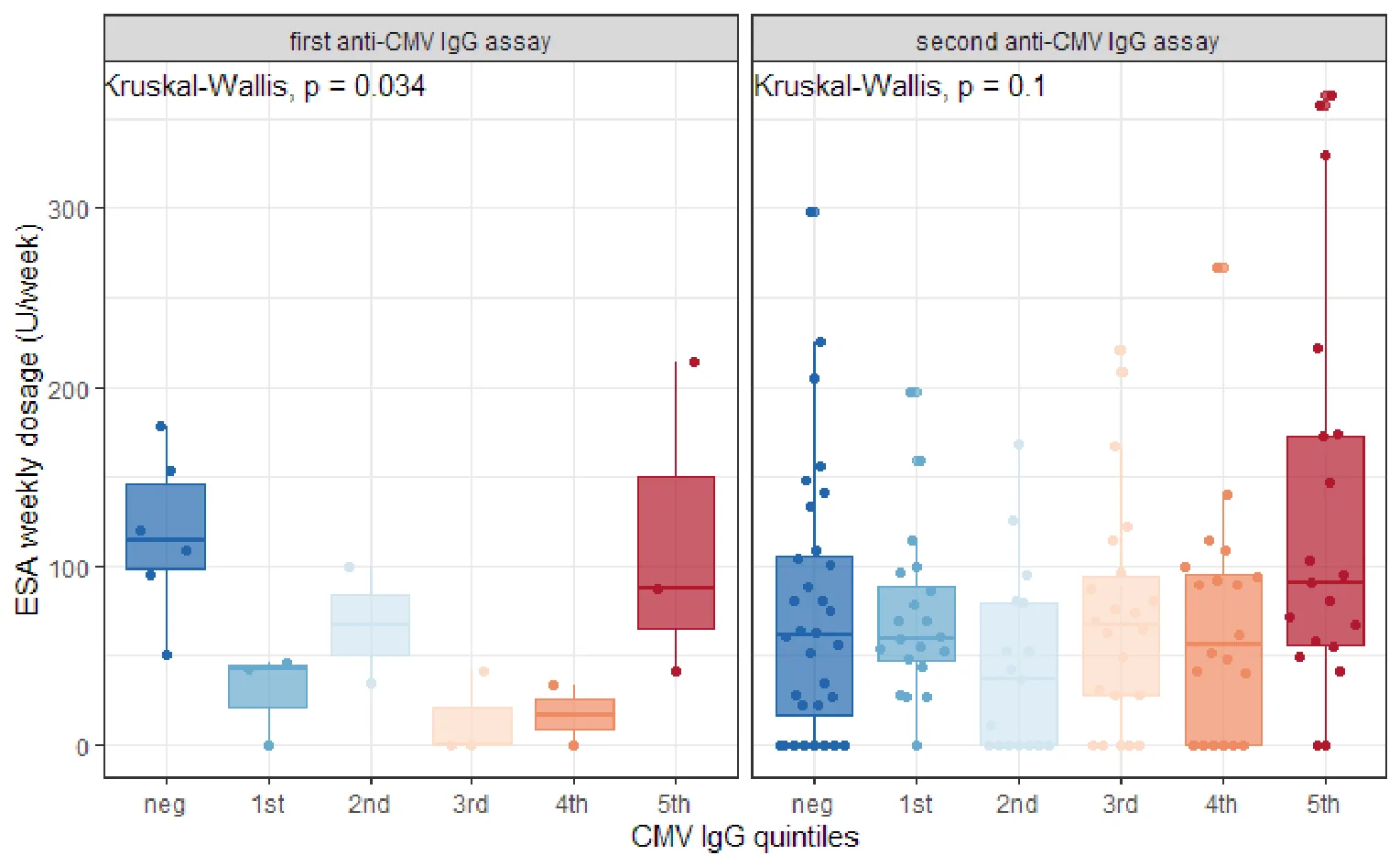
Erythropoiesis-stimulating agent (ESA) standardized weekly dose among patients with ESKD, stratified by CMV IgG quintiles, and faceted by the two anti-CMV IgG assays. Rates across multiple groups were compared using the Kruskal–Wallis test. ESKD = end-stage kidney disease; CMV = cytomegalovirus; IgG = immunoglobulin G.

In the adjusted pooled models (Table 3), individuals in the highest anti-CMV IgG quintile had significantly higher standardized weekly ESA requirements (increase of 63 units/week/kg, *p* = 0.005, *q* = 0.04), compared to the lowest quintile. Additionally, higher hemoglobin and CRP levels were associated with lower ESA dose. Figure 2 shows the predicted ESA weekly dose from the multivariate adjusted model, stratified by the presence of diabetes. In sensitivity analysis, the exclusion of influential individuals did not change the direction and statistical significance of the estimates (see Table A2). In addition, we modeled the Erythropoiesis Resistance Index (ERI), calculated as ESA weekly dosage per hemoglobin (g/dL), as in Betjes et al., which showed similar direction and significance of the highest anti-CMV IgG quintile, and hemoglobin and CRP levels (Table A3). Finally, we compared the models for the two anti-CMV IgG assays. The direction and magnitude were comparable to those from the initial model; however, they did not show statistical significance, likely due to limited sample size (Table A4, Figure A2).

## 4. Discussion

The present study examined the association between latent CMV infection, defined as presence of serum anti-CMV IgG, and anti-CMV IgG levels and both ESA prescription and weekly ESA dose in patients with ESKD.

The CMV seroprevalence of 79% in this cohort is consistent with reports from the general adult population [51,52] (40–94%) and from patients with ESKD [53,54,55,56] (77–98%). The prevalence of CMV latency increases with age and is associated with race, ethnicity, socioeconomic status, and education level [51]. As previously reported, women are more susceptible to CMV infection [16].

In this cohort, latent CMV infection was not associated with ESA prescription rate and hemoglobin levels. In contrast, both serum ferritin [57] and CRP [58,59] levels were higher, while TIBC levels were lower in CMV seropositive individuals, possibly reflecting higher age in this group. As a major limitation of the study, the levels of serum albumin, parathyroid hormone levels, use of angiotensin-converting enzyme (ACE) inhibitor or angiotensin receptor blocker (ARB), dialysis adequacy, residual renal function, and recent infections or blood loss were not available in this study. Importantly, data on the amount and frequency of any recent blood transfusion were not available.

In the adjusted regression model, ESA prescription was not associated with latent CMV infection; however, the lowest anti-CMV IgG quintile was associated with increased odds of ESA prescription. Also, in this adjusted model, diabetes was associated with increased odds, whereas atherosclerosis was associated with lower odds. We hypothesize that the comparative baseline (i.e., CMV seronegativity) is acting as a mediator in this model, significantly changing the associations. However, since we were only focused on CMV serostatus, and the number of individuals was small, we did not explore those associations further (with, for example, interaction terms). Although observational studies have shown that patients with polycystic kidney disease have relatively elevated erythropoietin production [43,44,45], ESA prescription was less frequent in unadjusted analysis only, and ESA weekly dose was not associated with this diagnosis in the present cohort.

Our results complement the findings from a comparable investigation by Baldeo et al., conducted in CKD patients prior to renal transplantation. This study found no association between CMV serostatus and epoetin dose, after adjustment for iron deficiency and ACE inhibitor/ARB use [49]. Those observations are in contrast with previous reports indicating that CMV infection may impair erythropoietin production and is associated with renal anemia [47], and that CMV seropositivity is associated with increased ESA requirements [48]. As our and Baldeo’s cohorts comprised patients being evaluated for future renal transplantation, they may carry a lower overall inflammatory burden, which could in turn affect ESA responsiveness. The discrepancy in outcomes can therefore be partly attributed to differences in the patient populations studied. Also, the selected population was defined on the basis that CMV serostatus is routinely available for renal transplantation candidates. This introduced a selection bias, as the included patients are generally healthier than the broader ESKD population. Consequently, the findings of this observational study may not be generalizable to all patients with ESKD.

While weekly ESA dose was not altered by CMV-serostatus alone, we observed higher weekly ESA doses in patients with anti-CMV IgG levels in the 5th quintile after adjustment for the important confounders. This observation supports the hypothesis that elevated serum anti-CMV Ig levels are associated with ESA hyporesponsiveness. Two potential explanations may account for this association. First, elevated anti-CMV IgG levels may be a marker of immune dysregulation, associated with low-level inflammation. It is well-established that systemic inflammation contributes to both atherosclerosis and increased ESA demand [46]. Won et al. [60] previously described a direct relationship between ESA dose and arterial microcalcifications in dialysis patents. Second, a limited number of studies suggest that elevated anti-CMV IgG levels are associated with CMV reactivations [42,61,62]. This leads to hypothesis that in individuals with elevated anti-CMV IgG, CMV reactivation might directly inhibit the erythropoietin production, thereby necessitating higher ESA doses, as shown in the work of Butler et al. [47].

Notably, our multivariate analysis revealed a weak, albeit significant, association between serum CRP levels and lower ESA requirements (Table 3), which is contrary to the expectations. However, CRP in this study is positively correlated with ferritin levels and negatively with hemoglobin levels; therefore this small and negative association might reflect confounding bias.

In addition to the major limitations stated above, this study utilized two different anti-CMV IgG assays that are not exchangeable and for which no established conversion function is available. Therefore, the quintile approach was employed, in which each patient’s result was categorized according to the distribution of values in each assay-specific group. While this directly resulted in loss of information, it also assumes comparable distribution between the two assay-specific groups. The smaller number of participants evaluated with the first assay may have additionally affected the comparability of these distributions, introducing an additional potential source of bias. In addition, the quintile approach resulted in small groups, thereby further limiting the generalizability of any observations. While observations were statistically significant, they remain hypothesis-generating.

The retrospective design represents a further and significant limitation. Although CMV seropositivity is typically asymptomatic and not related to the indication for testing, limiting inclusion to patients listed for renal transplantation introduced an important selection bias. The cohort also lacks racial diversity, reflecting the demographic characteristics of the national population. Furthermore, data on atherosclerosis were not collected systematically, and several important variables—such as diabetes type, prior immunomodulatory therapy, hyperlipidemia, and statin use—were unavailable.

## 5. Conclusions

The results of this study add to the evidence that anti-CMV IgG levels in the highest quintile are positively associated with higher standardized weekly ESA doses. Elevated CMV IgG levels may represent a marker of immune dysregulation, which is a recognized contributing factor to ESA hyporesponsiveness in ESKD. Alternatively, elevated anti-CMV IgG levels may be associated with subclinical CMV reactivation, potentially interfering with epoetin production. Whilst the present study did not investigate this effect, this warrants further research. Should such a causal association be confirmed, it would represent a novel and potentially treatable target for interventions aimed at reducing the high burden of anemia, ESA hyporesponsiveness, and inflammatory disbalance in patients with ESKD.

## Figures and Tables

**Table 1:** Baseline patients’ characteristics, stratified by CMV serostatus.

Characteristic	N	Overall N = 198 ^1^	CMV Seronegative N = 42 ^1^	CMV Seropositive N = 156 ^1^	*p*-Value ^2^
Age (years)	198	51 (38, 60)	44 (29, 53)	52 (40, 61)	0.004
Female	198	61 (31%)	9 (21%)	52 (33%)	0.14
Height (cm)	154	174 (167, 180)	177 (172, 180)	173 (167, 178)	0.090
Unknown		44	4	40	
Weight (kg)	154	74 (66, 84)	78 (72, 85)	73 (64, 84)	0.081
Unknown		44	4	40	
BMI (kg/m^2^)	154	24.5 (22.1, 27.9)	24.5 (22.6, 29.0)	24.5 (21.9, 27.7)	0.4
Unknown		44	4	40	
Kidney disease	198				
IgAN		43 (22%)	8 (19%)	35 (22%)	
Glomerulopathy		31 (16%)	7 (17%)	24 (15%)	
Diabetic		28 (14%)	4 (9.5%)	24 (15%)	
ADKPD		21 (11%)	1 (2.4%)	20 (13%)	
Genetic/congenital		11 (5.6%)	6 (14%)	5 (3.2%)	
Hypertensive		9 (4.5%)	1 (2.4%)	8 (5.1%)	
Obstructive		5 (2.5%)	4 (9.5%)	1 (0.6%)	
Other		17 (8.6%)	1 (2.4%)	16 (10%)	
Primary FSGS		7 (3.5%)	2 (4.8%)	5 (3.2%)	
Unknown		26 (13%)	8 (19%)	18 (12%)	
Diabetes	197	36 (18%)	4 (9.5%)	32 (21%)	0.10
Unknown		1	0	1	
Atherosclerosis	198	37 (19%)	6 (14%)	31 (20%)	0.4
Smoking	185	84 (45%)	13 (34%)	71 (48%)	0.12
Unknown		13	4	9	
RRT type	198				0.062
HD		145 (73%)	26 (62%)	119 (76%)	
PD		53 (27%)	16 (38%)	37 (24%)	
RRT vintage (years)	198	1.00 (0.30, 2.90)	0.55 (0.20, 1.30)	1.20 (0.40, 3.50)	0.006
ESA prescription	198	153 (77%)	32 (76%)	121 (78%)	0.9
ESA weekly dose (IU)	191	4500 (2000, 8000)	5500 (2000, 8000)	4000 (2000, 8000)	0.6
Unknown		7	0	7	
ESA weekly dose (IU/kg)	149	61 (27, 100)	70 (22, 120)	58 (27, 96)	0.4
Unknown		49	4	45	
Hemoglobin (g/L)	198	111 (104, 118)	111 (105, 115)	111 (104, 118)	0.6
CRP (mg/L)	196	4 (4, 8)	4 (4, 4)	4 (4, 10)	0.024
Unknown		2	1	1	
Ferritin (μg/L)	198	319 (176, 526)	238 (131, 428)	336 (187, 591)	0.027
TIBC (%)	194	45 (41, 49)	48 (44, 51)	44 (40, 48)	0.001
Unknown		4	1	3	
Iron (µmol/L)	198	11.1 (9.0, 13.5)	12.0 (9.6, 13.5)	11.0 (9.0, 13.5)	0.4

^1^ Median (Q1, Q3); n (%). ^2^ Wilcoxon rank sum test; Pearson’s Chi-squared test; NA. Abbreviations: ADPKD = autosomal dominant polycystic disease; BMI—body mass index; CMV = cytomegalovirus; ESA = erythropoiesis-stimulating agents; HD = hemodialysis; IgG = immunoglobulin G; RRT = renal replacement therapy; PD = peritoneal dialysis; CRP = C-reactive protein; TIBC = total iron-binding capacity.

**Table 2:** Baseline patients’ characteristics, stratified by anti-CMV IgG quintiles.

Characteristic	neg N = 42 ^1^	1st N = 30 ^1^	2nd N = 29 ^1^	3rd N = 30 ^1^	4th N = 29 ^1^	5th N = 30 ^1^	*p*-Value ^2^
Age (years)	44 (29, 53)	56 (37, 61)	44 (37, 55)	49 (42, 55)	56 (40, 62)	53 (43, 61)	0.039
Female	9 (21%)	9 (30%)	7 (24%)	11 (37%)	10 (34%)	12 (40%)	0.5
Weight (kg)	78 (72, 85)	70 (64, 77)	75 (68, 82)	72 (63, 82)	75 (64, 89)	72 (69, 82)	0.4
Unknown	4	6	9	3	6	8	
Height (cm)	177 (172, 180)	175 (169, 181)	175 (168, 177)	172 (166, 178)	169 (165, 174)	175 (168, 181)	0.14
Unknown	4	6	9	3	6	8	
BMI (kg/m^2^)	24.5 (22.6, 29.0)	22.8 (21.5, 25.4)	24.7 (22.2, 27.0)	24.2 (21.7, 27.5)	26.5 (22.7, 30.8)	24.1 (20.9, 26.3)	0.3
Unknown	4	6	9	3	6	8	
ADPKD	1 (2.4%)	4 (13%)	3 (10%)	5 (17%)	4 (14%)	4 (13%)	0.3
Diabetes	4 (9.5%)	4 (13%)	9 (31%)	2 (6.9%)	9 (31%)	6 (20%)	0.040
Unknown	0	0	0	1	0	0	
Atherosclerosis	6 (14%)	8 (27%)	4 (14%)	4 (13%)	6 (21%)	6 (20%)	0.7
Smoking	13 (34%)	10 (37%)	16 (57%)	13 (45%)	13 (45%)	16 (59%)	0.3
Unknown	4	3	1	1	0	3	
RRT type							0.7
HD	26 (62%)	23 (77%)	23 (79%)	22 (73%)	21 (72%)	22 (73%)	
PD	16 (38%)	7 (23%)	6 (21%)	8 (27%)	8 (28%)	8 (27%)	
RRT vintage (years)	0.55 (0.20, 1.30)	1.50 (0.50, 2.40)	1.00 (0.30, 2.00)	1.10 (0.60, 4.80)	1.60 (1.00, 4.80)	1.00 (0.30, 3.70)	0.009
ESA prescription	32 (76%)	27 (90%)	20 (69%)	22 (73%)	21 (72%)	26 (87%)	0.3
ESA weekly dose (IU)	5500 (2000, 8000)	4000 (3000, 6000)	2000 (0, 8000)	4000 (0, 6750)	4000 (0, 8000)	6000 (4000, 12,000)	0.072
Unknown	0	1	2	2	1	1	
ESA weekly dose (IU/kg)	70 (22, 120)	55 (43, 86)	37 (0, 80)	63 (0, 87)	49 (0, 94)	89 (55, 174)	0.056
Unknown	4	7	10	5	7	8	
Hemoglobin (g/L)	111 (105, 115)	112 (106, 118)	109 (103, 118)	110 (105, 121)	113 (104, 115)	112 (104, 120)	>0.9
CRP (mg/L)	4 (4, 4)	4 (4, 5)	4 (4, 9)	4 (4, 5)	4 (4, 7)	4 (4, 23)	0.2
Unknown	1	0	1	0	0	0	
Ferritin (μg/L)	238 (131, 428)	298 (171, 438)	427 (204, 700)	315 (205, 565)	389 (188, 622)	319 (161, 594)	0.2
TIBC	48 (44, 51)	46 (41, 48)	43 (40, 46)	43 (40, 49)	44 (39, 48)	45 (42, 49)	0.024
Unknown	1	1	0	0	0	1	
Iron (μmol/L)	12.0 (9.6, 13.5)	11.6 (8.9, 13.7)	11.8 (9.6, 12.8)	11.8 (10.0, 16.1)	9.2 (8.9, 11.0)	10.9 (9.0, 12.7)	0.12

^1^ Median (Q1, Q3); n (%). ^2^ Kruskal–Wallis rank sum test; Pearson’s Chi-squared test; Fisher’s exact test. Abbreviations: ADPKD = autosomal dominant polycystic disease; BMI—body mass index; CMV = cytomegalovirus; ESA = erythropoiesis-stimulating agents; HD = hemodialysis; IgG = immunoglobulin G; RRT = renal replacement therapy; PD = peritoneal dialysis; CRP = C-reactive protein; TIBC = total iron-binding capacity.

**Table 3:** Linear regression models predicting standardized ESA weekly dose (U/kg/week) (N = 148).

Characteristic	Univariate			Multivariate			
	Beta	95% CI	*p*-Value	Beta	95% CI	*p*-Value	q-Value ^1^
Age (years)	0.63	−0.42, 1.7	0.2	0.71	−0.37, 1.8	0.2	0.4
Female	19	−13, 51	0.2	3.9	−25, 33	0.8	0.8
Diabetes	−23	−59, 12	0.2	−28	−64, 7.0	0.11	0.3
Atherosclerosis	10	−27, 47	0.6	18	−20, 57	0.3	0.6
Smoking	4.6	−25, 34	0.8	7.1	−20, 35	0.6	0.8
RRT vintage (years)	−0.09	−3.8, 3.6	>0.9	−1.2	−5.1, 2.7	0.6	0.8
RRT type							
HD	—	—		—	—		
PD	3.2	−28, 34	0.8	5.1	−25, 35	0.7	0.8
ADPKD	−30	−68, 8.3	0.12	−6.8	−45, 31	0.7	0.8
Hemoglobin (g/L)	−1.9	−3.3, −0.55	0.007	−2.4	−3.9, −1.0	0.001	0.017
CRP (mg/L)	−0.09	−0.51, 0.33	0.7	−0.46	−0.88, −0.03	0.036	0.2
Ferritin (μg/L)	0.03	−0.03, 0.08	0.3	0.04	−0.01, 0.10	0.13	0.3
Anti-CMV IgG quintiles							
1st	—	—		—	—		
2nd	−35	−76, 6.3	0.10	−29	−68, 9.6	0.14	0.3
3rd	−11	−52, 29	0.6	−2.7	−40, 35	0.9	0.9
4th	−16	−58, 25	0.4	−12	−51, 27	0.5	0.8
5th	52	5.3, 98	0.029	63	19, 106	0.005	0.040

^1^ False discovery rate correction for multiple testing. Abbreviation: CI = confidence interval. Abbreviations: ADPKD = autosomal dominant polycystic disease; CI = confidence interval; CMV = cytomegalovirus; ESA = erythropoiesis-stimulating agents; IgG = immunoglobulin G; RRT = renal replacement therapy; HD = hemodialysis; PD = peritoneal dialysis.

## Data Availability

The datasets generated and/or analyzed during the current study are not publicly available due to restrictions related to personal data protection. Data were analyzed in pseudonymized form and are available from the corresponding author on reasonable request.

## Abbreviations

The following abbreviations are used in this manuscript:

ADPKD	Autosomal dominant kidney polycystic disease
BMI	Body mass index
CERA	Continuous epoetin receptor activator
CI	Confidence interval
CKD	Chronic kidney disease
CMV	Human cytomegalovirus
CRP	C-reactive protein
ESKD	End-stage kidney disease
ESA	Erythropoiesis-stimulating agents
eGFR	Estimated glomerular filtration rate
FSGS	Focal segmental glomerulosclerosis
HD	Hemodialysis
IgAN	IgA nephropathy
IgG	Immunoglobulin G
OR	Odds ratio
PD	Peritoneal dialysis

## Appendix A

**Table A1:** Regression models predicting ESA prescription rate.

Characteristic	Univariate					Multivariate–CMV Serostatus				Multivariate-CMV IgG Quintiles			
	N	OR	95% CI	*p*-Value	q-Value ^1^	OR	95% CI	*p*-Value	q-Value ^1^	OR	95% CI	*p*-Value	q-Value ^1^
Female	198	0.76	0.38, 1.55	0.4	0.7	0.48	0.21, 1.12	0.089	0.2	0.40	0.16, 0.99	0.049	0.2
CMV serostatus	198	1.08	0.47, 2.35	0.9	0.9	1.37	0.53, 3.41	0.5	0.7				
Age (years)	198	0.99	0.97, 1.01	0.5	0.7	1.01	0.98, 1.03	0.7	0.8	1.01	0.98, 1.04	0.6	0.7
Atherosclerosis	198	0.54	0.25, 1.21	0.12	0.6	0.31	0.11, 0.89	0.030	0.2	0.23	0.07, 0.70	0.011	0.091
Diabetes	198	1.59	0.65, 4.47	0.3	0.7	2.94	0.99, 10.3	0.067	0.2	4.25	1.33, 16.1	0.022	0.12
Smoking	198	1.27	0.65, 2.52	0.5	0.7	1.01	0.45, 2.26	>0.9	>0.9	0.92	0.39, 2.18	0.9	0.9
RRT vintage (years)	198	1.01	0.93, 1.13	0.8	0.8	1.09	0.98, 1.24	0.2	0.3	1.09	0.97, 1.25	0.2	0.3
RRT type	198												
HD		—	—			—	—			—	—		
PD		1.37	0.64, 3.13	0.4	0.7	2.32	0.92, 6.33	0.085	0.2	2.58	0.97, 7.55	0.069	0.2
ADPKD	198	0.34	0.13, 0.89	0.024	0.2	0.61	0.20, 2.03	0.4	0.6	0.51	0.15, 1.76	0.3	0.5
Hemoglobin (g/L)	198	0.96	0.93, 0.99	0.014	0.2	0.93	0.89, 0.97	0.002	0.019	0.92	0.87, 0.96	<0.001	0.006
CRP (mg/L)	198	0.99	0.98, 1.00	0.15	0.6	0.99	0.98, 1.00	0.10	0.2	0.99	0.98, 1.00	0.084	0.2
Ferritin (μg/L)	198	1.00	1.00, 1.00	0.8	0.8	1.00	1.00, 1.00	0.6	0.7	1.00	1.00, 1.00	0.8	0.9
tibc	198	0.97	0.93, 1.02	0.3	0.7								
Iron	198	1.04	0.95, 1.14	0.4	0.7								
Anti-CMV IgG quintiles	198												
neg		—	—							—	—		
1st		2.81	0.77, 13.5	0.14	0.6					4.97	1.14, 28.1	0.045	0.2
2nd		0.69	0.24, 2.03	0.5	0.7					0.66	0.19, 2.32	0.5	0.7
3rd		0.86	0.29, 2.58	0.8	0.8					1.29	0.37, 4.68	0.7	0.8
4th		0.82	0.28, 2.47	0.7	0.8					0.76	0.22, 2.70	0.7	0.8
5th		2.03	0.60, 8.09	0.3	0.7					4.16	0.99, 21.0	0.063	0.2
NA		0.52	0.11, 2.89	0.4	0.7					0.37	0.05, 2.82	0.3	0.5

^1^ False discovery rate correction for multiple testing. Abbreviations: CI = confidence interval, OR = odds ratio.

**Table A2:** Sensitivity analysis for model predicting standardized ESA weekly dose: pooled linear regression model from the full imputed dataset compared to the linear regression model on trimmed dataset, in which the outliers were removed (see Methods).

Term	Estimate— Full Dataset	Estimate— Trimmed Dataset	*p*-Value—Full Dataset	*p*-Value— Trimmed Dataset
(Intercept)	284.461	272.316	0.000	0.000
Age (years)	0.738	0.399	0.158	0.363
Female	3.451	−11.666	0.818	0.376
Diabetes	−29.799	−15.445	0.104	0.321
Atherosclerosis	18.551	10.891	0.369	0.578
Smoking	8.127	5.780	0.535	0.596
RRT vintage (years)	−0.810	−0.475	0.727	0.829
RRT type				
PD	4.223	−12.391	0.772	0.331
ADPKD	−3.586	1.203	0.857	0.943
Hemoglobin (g/L)	−2.388	−2.017	0.001	0.001
CRP (mg/L)	−0.485	−0.402	0.042	0.050
ferritin (μg/L)	0.040	0.027	0.196	0.271
Anti-CMV IgG quintiles				
igg.quartiles2nd	−25.441	−33.517	0.252	0.077
igg.quartiles3rd	1.664	−0.812	0.931	0.959
igg.quartiles4th	−9.402	−16.884	0.645	0.313
igg.quartiles5th	69.563	43.457	0.001	0.015

**Table A3:** Linear regression models predicting standardized ESA weekly dose per hemoglobin (N = 148).

Characteristic	Univariate			Multivariate			
	Beta	95% CI	*p*-Value	Beta	95% CI	*p*-Value	q-Value ^1^
Age (years)	0.05	−0.05, 0.15	0.3	0.05	−0.06, 0.15	0.4	0.8
Female	1.9	−0.99, 4.7	0.2	1.5	−1.3, 4.3	0.3	0.8
Diabetes	−1.3	−5.0, 2.4	0.5	−2.1	−6.1, 1.9	0.3	0.8
Atherosclerosis	0.30	−3.2, 3.8	0.9	0.57	−3.3, 4.5	0.8	>0.9
Smoking	−0.05	−2.8, 2.7	>0.9	0.06	−2.6, 2.7	>0.9	>0.9
RRT vintage (years)	0.02	−0.38, 0.41	>0.9	−0.12	−0.56, 0.31	0.6	>0.9
RRT type							
HD	—	—		—	—		
PD	−0.10	−2.9, 2.7	>0.9	−0.68	−3.6, 2.3	0.6	>0.9
ADPKD	−1.6	−5.5, 2.2	0.4	−1.8	−6.0, 2.4	0.4	0.8
CRP (mg/L)	−0.01	−0.05, 0.03	0.6	−0.02	−0.06, 0.02	0.3	0.8
Ferritin (μg/L)	0.00	0.00, 0.01	0.5	0.00	0.00, 0.01	0.3	0.8
Anti-CMV IgG quintiles							
1st	—	—		—	—		
2nd	−1.8	−6.0, 2.4	0.4	−1.2	−5.4, 3.1	0.6	>0.9
3rd	−0.51	−4.5, 3.5	0.8	−0.03	−4.0, 4.0	>0.9	>0.9
4th	−0.94	−4.9, 3.0	0.6	−0.45	−4.5, 3.6	0.8	>0.9
5th	5.4	1.2, 9.5	0.013	5.8	1.7, 10	0.006	0.086

^1^ False discovery rate correction for multiple testing. Abbreviation: CI = confidence interval.

**Table A4:** Sensitivity analysis for model predicting standardized ESA weekly dose: models predicting ESA weekly dose with both assays for measuring anti-CMV IgG.

	First Anti-CMV IgG Assay					Second Anti-CMV IgG Assay				
Characteristic	N	Beta	95% CI	*p*-Value	q-Value ^1^	N	Beta	95% CI	*p*-Value	q-Value ^1^
Age (years)	23	−0.52	−7.9, 6.8	0.9	>0.9	125	0.50	−0.56, 1.5	0.4	0.8
Female	23	41	−188, 269	0.7	>0.9	125	−0.03	−32, 32	>0.9	>0.9
Diabetes	23	−7.1	−279, 265	>0.9	>0.9	125	−35	−75, 4.5	0.081	0.4
Atherosclerosis	23	−60	−211, 90	0.3	>0.9	125	32	−9.1, 74	0.12	0.5
Smoking	23	83	−139, 304	0.3	>0.9	125	7.9	−23, 39	0.6	>0.9
RRT vintage (years)	23	1.9	−34, 38	0.9	>0.9	125	−0.46	−7.2, 6.3	0.9	>0.9
RRT type	23					125				
HD		—	—				—	—		
PD		−77	−459, 304	0.6	>0.9		4.8	−29, 39	0.8	>0.9
ADPKD	23	29	−266, 325	0.8	>0.9	125	−12	−53, 30	0.6	>0.9
Hemoglobin (g/L)	23	3.2	−18, 24	0.7	>0.9	125	−2.3	−3.7, −0.85	0.002	0.030
CRP (mg/L)	23	−0.26	−1.9, 1.4	0.7	>0.9	125	−0.43	−1.2, 0.30	0.2	0.6
Ferritin (μg/L)	23	0.05	−0.15, 0.26	0.5	>0.9	125	0.02	−0.04, 0.09	0.4	0.8
Anti-CMV IgG quintiles	23					125				
1st		—	—				—	—		
2nd		42	−89, 173	0.5	>0.9		−29	−74, 15	0.2	0.6
3rd		29	−132, 191	0.6	>0.9		3.9	−40, 47	0.9	>0.9
4th		127	−266, 520	0.4	>0.9		−5.8	−50, 39	0.8	>0.9
5th		45	−88, 179	0.4	>0.9		62	13, 110	0.014	0.10

^1^ False discovery rate correction for multiple testing. Abbreviation: CI = confidence interval.
